# Emerging insect-based aquafeed for sustainable African catfish production

**DOI:** 10.1371/journal.pone.0335422

**Published:** 2025-11-26

**Authors:** Anderson N. Maina, Isaac M. Osuga, Leonard K. Munga, Jonathan M. Munguti, Subramanian Sevgan, Didier K. Barwani, Menaga Meenakshisundaram, Jimmy B. Mboya, Stavroula Bourou, Aikaterini Zachariadi, Vassilios Zachariadis, Rodrigue Yossa, Dennis Beesigamukama, Brian O. Ochieng, Abdullahi A. Yusuf, Shaphan Y. Chia, David M. Liti, Chrysantus M. Tanga

**Affiliations:** 1 International Centre of Insect Physiology and Ecology (icipe), Nairobi, Kenya; 2 Department of Animal Science, Kenyatta University, Nairobi, Kenya; 3 Department of Animal Sciences, Jomo Kenyatta University of Agriculture and Technology, Nairobi, Kenya; 4 Kenya Marine and Fisheries Research Institute, Sagana, Kenya; 5 Department of Animal Production, Université de Kalemie (UNIKAL), DR Congo; 6 Department of Agriculture Development, National and Kapodistrian University of Athens, Athens, Greece; 7 WorldFish, Bayan Lepas, Penang, Malaysia; 8 Department of Zoology and Entomology, University of Pretoria, Pretoria, South Africa; 9 Forestry and Agricultural Biotechnology Institute, University of Pretoria, Pretoria, South Africa; 10 Department of Biological Sciences, University of Eldoret, Eldoret, Kenya; Sher-e-Kashmir University of Agricultural Sciences and Technology of Kashmir, INDIA

## Abstract

The current study assessed the implications of substituting fish meal (FM) with black soldier fly larvae meal (BSFLM) on sustainable African catfish (*Clarius gariepinus*) production. Five isocaloric experimental diets were formulated and 168-days feeding trials were performed with 1400 improved strains of African catfish. Fish fed with 50% BSFLM expressed the lowest and highest feed conversion ratio, and daily weight gain and specific growth rate. Fillets from fish fed diet with 50 and 75% BSFLM inclusion expressed higher lauric (11.4–16.7%) and linoleic acids. Omega-6 fatty acid was 19-fold higher in fillets from fish fed diet with 75%BSFLM. There was 5–8% and 9–11% higher crude protein in fillet from fish fed diet with 50 and 75% BSFLM, respectively. Essential amino acids in the fillets, particularly lysine [11 – 18g/kg] and methionine [11 – 27g/kg] values were significantly higher in catfish fed diet with BSFLM diets than with FM. Diet integrated with 50% BSFLM showed the best return on investment (105.2%) and cost-benefit ratio (1.05). Integration of 50–75% BSFLM into African catfish diet offers a sustainable and cost-effective alternative to conventional fish feed, with promising long-term nutritional and health benefits for the aquaculture industry.

## Introduction

The current spiralling of the world human populace is leading to a heightened rate of animal protein consumption, with a particular emphasis on fish [1,2]. The rising demand for protein underscores the importance of exploring alternative sources of animal protein, such as insect meals, particularly black soldier fly larvae meal (BSFLM). This exploration, in turn, contributes to increased fish production and a concomitant reduction in production costs to satisfy the escalating demand for animal sourced proteins [2,3]. Furthermore, adoption of insect meals has been reported to reduce overreliance on fishmeal (FM), the aquacultural primary sources of protein [4,5] chiefly sourced from seas, oceans, and inland waterways, which have often been prohibitively costly, leading to expensive fish feeds. Additionally, the need to sustainably utilize marine resources and reduce human-livestock food-feed competition necessitates a rethink on alternative animal protein sources [6–8].

Black soldier fly larvae (BSFL) can be mass-produced cheaply from various organic substrates [9]. In addition, their ability to degrade organic waste has the potential for use in waste management [10,11], resulting in a protein-rich product that can be processed into a meal for use by livestock [12,13]. The BSFLM has satisfactorily been embraced in multiple studies as a principal source of protein in poultry, swine, and other species of livestock with encouraging results [14–20]. Furthermore, dietary formulations containing BSFLM have undergone evaluation across diverse species of fish, encompassing channel catfish [21], Nile tilapia [22–24], rainbow trout [25–29], pikeperch [30], turbot [31], hybrid tilapia [32] and Atlantic salmon [33–36]. These investigations consistently indicate that BSFLM can partially substitute FM in fish diets, without impairing health indices and growth performances. Zhou et al. [37] and Li et al. [38] documented that the incorporation of BSFLM did not yield detrimental effects on the health, quality parameters of carcass, biological characteristics, and growth of Jian carp (*Cyprinus carpio var.* Jian). In addition, Stamer et al. [39] noted better performance in rainbow trout reared on BSFLM-based diets compared to those fed on fishmeal-based diets. Xiao et al. [40] also reported favorable results while utilizing BSFLM in raising the yellow catfish (*Pelteobagrus fulvidraco*). Zebrafish (*Danio rerio*) partially reared on BSFL meal exhibited no adverse impact on gut and histomorphology indices and may potentiate the growth factor genes [41].

Numerous studies have examined the effects of substituting fish meal (FM) with black soldier fly larvae meal (BSFLM) in the diets of *Clarias gariepinus*, with findings supporting BSFLM as a promising alternative to FM [42–47]. However, most of these studies have primarily centered on the growth performance of *C. gariepinus* fed BSFLM, typically over short culture periods at the fry and fingerling stages. Notably, only Gebremichael et al. [45] investigated the fillet properties of *C. gariepinus* after extended feeding with insect meal-integrated diets, including BSFLM and mealworm meal. Nevertheless, this study did not explore different incorporation levels of BSFLM as a substitute to FM, and it utilized defatted BSFLM, which affected the fillet quality of the fish.

In aquaculture, besides the growth performance and nutritional quality implications of a diet, it is vital to consider its economic advantage over other alternatives. When selecting ingredients for feed formulation, it is essential to consider not only a balanced dietary source to ensure optimal growth but also economic factors, such as cost-effectiveness [24,47,48]. Previous studies have explored the economic implications of replacing FM with BSFLM in *C. gariepinus* diet. Maranga et al. [47] found that substituting dietary FM with up to 75% fully defatted BSFLM can substantially reduce feed cost and increase profit margin in *C. gariepinus* production. This can be ascribed to the cost of BSFLM production, which is comparatively lower in tropical regions as compared to temperate regions [5].

Consequently, the present study sought to investigate the implications of varying levels of inclusion of full-fat- BSFLM on growth performance and fillet nutritional quality of *C. gariepinus*, and on the cost-effectiveness of its production for a prolonged culture period. The findings of this study are vital for improving the yield and nutritional value of *C. gariepinus*, as well as the economic sustainability of *C. gariepinus* production through use of sustainable alternative sources of dietary protein.

## Materials and methods

### Ethics statement

This study was performed in accordance with ARRIVE Guidelines, which is consistent with the Ethical Principles in Animal Research adopted by the Institutional Animal Care and Use Committee (IACUC) of the Veterinary Science Research Institute (VSRI) of Kenya Agricultural and Livestock Research Organization (KALRO), with application number: KALRO-VSRI/IACUC028/16032022 and was approved by the National Commission for Science, Technology, and Innovation (NACOSTI), with research permit number NACOSTI/P/20/6955. Following endorsement by the IACUC and NACOSTI, Kenyatta University [KU] was informed about the objectives of the study through a support letter. After reviewing the proposal, KU wrote a permission and support letter to the Kenya Marine and Fisheries Research Institute (KMFRI), Sagana, Kirinyaga County. Then, the lead author of this paper and staff of KMFRI selected the fish for the present study. All experimental procedures involving fish were performed in accordance with the guidelines of IACUC of VSRI. The frequency of fish monitoring during the growth and survival stages were regular to observe the fish condition.

### Study design on diets

The research was executed at the Sagana Centre-based Kenya Marine and Fisheries Research Institute (KMFRI), situated at latitudes 0019’S and 37012’E. Full-fat black soldier fly larvae (BSFL) reared on market waste (vegetables and fruits) were procured from the International Center of Insect Physiology and Ecology (*icipe*), Nairobi, oven dried, and kept in sealed bags at normal room temperature (25˚C) conditions until future utilization. The other ingredients were obtained from the local suppliers, processed, blended, and compounded to satisfy the optimum dietary needs of the African Catfish [49,50]. All the ingredients were ground using a Lab Universal Grinder (ZX, Model FW200), individually weighed (WSS S.S Waterproof Baker Dough Scale, Model WSS6), and mixed with a laboratory blender (Waring LBC15 Stainless Steel Laboratory Blender, 1 gal, 120 V/60 Hz). The five experimental diets were formulated to achieve isoenergetic (20 KJ available energy g^-1^ of diet), isonitrogenous (40% crude protein, as fed), and isolipidic (12% crude fat) diet. This was accomplished through the application of linear least-cost formulation software (WinFeed Limited, Cambridge, UK), with the diets structured in graded series to facilitate the complete or partial substitution of FM with BSFLM. Five experimental diets were developed by replacing FM with full-fat BSFLM at varying levels as 0 percent BSFLM and 100 percent FM (C – control diet), 25 percent BSFLM and 75 percent FM (D1), 50 percent BSFLM and 50 percent FM (D2), 75 percent BSFLM and 25 percent FM (D3), 100 percent BSFLM and 0 percent FM (D4), and analyzed for their proximate composition to ensure the required nutrient composition. The formulated experimental diets were transferred into and sealed in polyethylene bags and stored at 25°C for later utilization in the experiment. The fatty acid profiles of the full-fat BSFL, fish meal and wheat bran are provided in Table 1.

**Table 1:** Fatty acids content (mg/g) of full fatted BSFLM in the current study [in parentheses] compared to range of values documented fishmeal and wheat bran in literature.

Fatty acid	Full-fat BSFL	Fish meal	Wheat bran
C10:0	0.0015	–	–
C11:0	0.0003	–	–
C12:0	0.1639	–	–
Iso-dimethyl-C11:0	0.0007	–	–
Iso-methyl-C12:0	0.0039	–	–
C13:0	0.0020	–	–
Iso-methyl-C13:0	0.0032	–	–
Iso-methyl-C13:0	0.0270	–	–
C14:0	0.1274	0.06	–
Iso-methyl-C14:0	0.0113	–	–
Iso-methyl-C14:0	0.0134	–	–
Iso-methyl-C14:0	0.0052	–	–
Iso-methyl-C15:0	0.0063	–	–
C16:0	0.0755	0.23	0.19
Iso-methyl-C16:0	0.0076	–	–
Iso-methyl-C17:0	0.0152	–	–
Iso-methyl-C16:0	0.0167	–	–
Iso-methyl-C16:0	0.0039	–	–
C18:0	0.0663	–	–
Iso-methyl-C18:0	0.0227	–	–
C20:0	0.0211	–	–
Iso-methyl-C19:0	0.0171	–	–
C22:0	0.0129	–	–
C24:0	0.0082	–	–
Iso-methyl-C18:1(n-9)	0.0077	–	–
C10:1(n-5)	0.0019	–	–
C16:1(n-7)	0.0258	0.09	–
C18:1(n-9)	0.0969	0.16	0.40
C22:1(n-9)	–	0.04	–
C24:1(n-9)	–	0.007	–
C18:2(n-2)	0.0009	–	–
C18:2(n-6)	0.0577	–	–
C18:3(n-3)	11.3092	–	0.03
C18:4(n-3)	0.6287	–	–
C20:5(n-3)	0.3117	0.08	–
C22:6(n-3)	0.2123	0.16	–
C18:2(n-7)	0.0212	–	–

### Sample size of experimental fish and procedures

The study deployed a completely randomized design (CRD) featuring five distinct treatments in three replications each. A total of 1400 Sagana (improved through selective breeding) strain of African catfish, exhibiting uniform sizes (approximately 3.5–4.0 grams), were obtained from KMFRI Sagana Centre. Before initiating the feeding experiment, the catfish were acclimatized for one week. Later, the fish were stocked at a density of 10 fish/m^3^ in fixed net cages in pond (2x2x2m) in triplicates per treatment. The cages were installed in an earthen pond (800m^3^) and monitored for 168 days. The fish were fed two times per day at 9:00 am in the morning and 3:00 pm in the evening, with feed rations being adjusted based on weight measurements, ranging from 2–8% of the total biomass throughout the study period. The feed to be administered in each cage was weighed daily using a digital weighing balance (Tanita KD 200 digital scale, precision: 0-1000g x 1g). The overall care and management of the experimental fish adhered to the guidelines outlined by McGlone [51] and were supervised by the aquaculture technical staff at KMFRI.

### Measurement of physico-chemical water quality parameters

Water sampling for the experiment followed the standard guidelines outlined by the American Public Health Association [52]. Daily monitoring included the measurement of dissolved oxygen (mg/l), pH, conductivity (mho cm^-1^), total dissolved solids (TDS) (mg/l), temperature (°C) at 8:00 am before feeding with the help of a YSI multi-parameter water quality meter (Am Achalaich 11, 82362 Weilheim, Germany). To determine levels of nitrogen forms of ammonia, nitrate and phosphorus, 100 ml of water was collected weekly and analyzed in the laboratory using a UV-1800 Shimadzu spectrometer (Shimadzu Corporation, Tokyo, Japan). Corresponding phosphorus and nitrogen (µmoles/l) concentration were calculated from the amounts as mg/l of phosphate (PO_4_), nitrate-nitrogen (NH_4_-N) and nitrite-nitrogen (NO_3_-N) in the water.

### Measurement of growth and survival of fish fed on different feed types

The growth performance was assessed through weekly sampling, where the fish were randomly collected from the cages and shipped in water-filled buckets to reduce stress and minimize potential mortalities. Fifty (50) catfish from each cage were weighed using a digital weighing balance (Tanita KD 200 digital scale, precision: 0-1000g x 1g), and their lengths taken using a standardized meter rule. Percentage survival rates were calculated at the conclusion of the experiment by calculating the percentage of surviving fish in each treatment. The weight gain (g), feed conversion ratio (FCR), specific growth rate (SGR; %) and survival rate (%) were calculated from the primary data using equations 1, 2, 3 and 4 respectively. At the termination of the trial, fish from every cage were euthanized by immersing in ice for four minutes, sampled and temporarily kept at −20 °C for further analyses.


$$\text{Weight gain}=\text{Final mean weight}(W_{1})-\text{initial mean weight}(W_{0})$$ (1)

$$\text{Feed Conversion Ratio}(\text{FCR})=\frac{\text{Average feed intake}(\text{g})}{\text{Mean weight gain}(\text{g})}$$ (2)

$$\text{Specific Growth Rate}(\text{SGR})=\frac{[\text{n}(W_{1})-\text{ln}(W_{0})]}{\text{time in days}}\text{x 100}$$ (3)

where:

1. ln (W_1_) is the natural log of the final weight,
2. ln (W_0_) is the natural log of the initial weight,

$$\text{Survival rate}(\%)=\frac{\text{Final number of fish}}{\text{Initial number of fish}}\times 100$$ (4)

### Determination of proximate composition of fish diets and fish fillet quality

The proximate composition of the experimental diets samples and catfish fillets were assessed in accordance with the protocols outlined by Thiex [53]. The ash contents were determined through an 8-hour ashing process at 550 °C. Crude fat was estimated in a solvent extractor (VELP Scientifica, Usmate (MB), Italy) using ethyl ether as the extracting solvent set at a boiling point of 60°C. Crude protein levels were estimated by computing the product of a 6.25 conversion factor and nitrogen content (% N) obtained from the Kjeldahl machine [54,55].

### Determination of amino acid and fatty acid composition of fish fillet

Experimental fish fillets were analyzed for amino acid composition by adopting the methods of Musundire et al. [56] and Hamilton et al. [57]. About 25 mg of powdered fish fillet were dampened with 5 mL of 6N HCl in Pyrex tubes. Upon introduction of nitrogen gas, the tubes were stoppered and the contents hydrolyzed in an oven at 110°C for 22 hours. Hydrolysates in each Pyrex tube were dried and then reconstituted with 1 mL 0.01% formic acid/acetonitrile (95: 5), agitates vigorously, centrifuged and the supernatant (0.3 *µL*) injected into a liquid chromatography-mass spectrometry (LC-MS) to separate, identify, and quantify amino acids.

Modified methods of and instrument operating conditions outlined in Ochieng et al. [58] were considered for the transformation of fatty acids to fatty acids methyl esters (FAMEs) and their subsequent identification and quantitative analysis in the Gas Chromatography Mass Spectrometry (GC-MS). Briefly, 100 mg of the ground fish fillet were trans-methylated using methanolic sodium methoxide solution. The generated FAMEs were extracted from the mixture by addition of 1 mL of hexane, centrifuged and the supernatant analyzed in the GC-MS after drying over anhydrous sodium sulphate. The GC-MS comprised of a 7890A gas chromatograph connected to a 5975 C mass selective detector (Agilent Technologies Inc., Santa Clara, CA, USA).

### Cost-benefit analysis of fish production on various diets

Economic assessments of dietary replacement of FM with BSFL meal in the African catfish diets were conducted through the calculation of return on investment (RoI) and cost-benefit ratio (CBR) using equations 5, 6, 7, and 8 [48,59,60]. Feed costs were determined based on ingredient costs, where the cost of FM was USD 1.60 per Kg, while the cost of dried full-fat BSFLM was USD 1.25 per Kg. The prevailing market price for a Kg of African catfish was USD 2.50. A CBR value exceeding 1 indicated that the advantages of replacing FM with the whole BSFL meal surpassed the associated production costs. Therefore, the cost of the consumed feed was assumed to be the only price source derived from the production process.


GrossProfit=Revenuefromsaleofharvestedfish−Totalcostofproduction (5)

$$Grossprofitmargin=\frac{Revenuefromsaleofharvestedfish-Totalcostofproduction}{Revenuefromsaleofharvestedfish}$$ (6)

$$CBR=\frac{Totalcostofproduction}{Revenuefromsaleofharvestedfish}$$ (7)

$$RoI=\frac{Grossprofit}{Totalcostofproduction}\times 100\%$$ (8)

### Statistical analysis

All data analysis were performed using R Studio software version 1.3.1093–1 [61] for Windows at α = 5%. Shapiro-Wilk test and Box plots (*P* < 0.05) were adopted for data exploration to ascertain normal distribution of the data. For the comparison of growth performance, fatty acids (FAs), and amino acids (AAs) composition among experimental diets, a one-way ANOVA test, followed by Tukey’s test was performed.

## Results

### Feed ingredients and diet composition

The diet composition and analyzed proximate parameters are indicated in Table 2. All the diets were developed to satisfy the nutritional requirements of African catfish.

**Table 2:** Experimental diets formulations and their respective nutrient quality.

Experimental Diets					
Ingredient	C	D1	D2	D3	D4
Rice bran	10.00	10.00	10.00	10.00	10.00
Corn gluten meal	10.00	10.00	10.00	10.00	10.00
Corn (7.5% CP)	22.06	15.39	07.80	03.86	01.15
Wheat bran	05.00	06.01	07.94	06.22	03.28
BSFL	0.00	17.12	34.24	51.36	68.47
Fishmeal (FM)	45.84	34.38	22.92	11.46	0.00
Canola oil	1.50	1.50	1.50	1.50	1.50
Fish oil	1.25	1.25	1.25	1.25	1.25
Dicalcium phosphate	2.00	2.00	2.00	2.00	2.00
Trace Mineral premix	1.00	1.00	1.00	1.00	1.00
Vitamin-Mineral Premix	0.45	0.45	0.45	0.45	0.45
DL-Methionine	0.20	0.20	0.20	0.20	0.20
L-Glutamic acid	0.50	0.50	0.50	0.50	0.50
L-Lysine	0.20	0.20	0.20	0.20	0.20
**Total**	**100.00**	**100.00**	**100.00**	**100.00**	**100.00**
**Analysed provisions on a dry matter basis (%)**					
Dry Matter (DM)	89.60 ± 0.06	89.63 ± 0.09	89.73 ± 0.09	89.77 ± 0.10	89.93 ± 0.10
Crude Protein (CP)	40.07 ± 0.09	40.07 ± 0.12	40.23 ± 0.11	40.00 ± 0.12	40.13 ± 0.15
Crude Fat (EE)	12.00 ± 0.06	12.07 ± 0.09	12.13 ± 0.12	12.50 ± 0.17	12.85 ± 0.10
Crude Fibre (CF)	6.03 ± 0.12	6.43 ± 0.15	6.93 ± 0.15	7.40 ± 0.20	7.93 ± 0.15
Ash	12.93 ± 0.12	12.83 ± 0.15	12.57 ± 0.15	12.43 ± 0.15	12.23 ± 0.15
NFE*	29.33 ± 0.11	28.63 ± 0.12	28.13 ± 0.10	28.23 ± 0.11	27.63 ± 0.12
Gross Energy (kcal/kg)	4702 ± 1.11	4715 ± 1.12	4735 ± 1.15	4762 ± 1.12	4795 ± 1.12

Note: *NFE-Nitrogen Free Extract. Ingredients composition (g/kg, as fed): Rice bran: Crude Protein (%):12.6 ± 0.03,Crude Fat (%): 17.2 ± 0.01,Crude Fiber (%): 7.15 ± 0.01,Crude Ash (%): 9.10 ± 0.10; Corn gluten meal: Crude Protein (%): 71.4 ± 0.01, Crude Fat (%): 4.1 ± 0.01,Crude Fiber (%): 0.8 ± 0.01,Crude Ash (%):1.2 ± 0.01; Corn: Crude Protein (%):7.5 ± 0.001,Crude Fat(%): 3.8 ± 0.001,Crude Fiber (%):2.00 ± 0.01,Crude Ash (%):1.2 ± 0.00; Wheat Bran: Crude Protein (%): 11.32 ± 0.19,Crude Fat(%):2.12 ± 0.04,Crude Fiber (%):7.53 ± 0.98,Crude Ash (%):3.90 ± 0.15; Black soldier fly larvae: Crude Protein (%):48.20 ± 0.05,Crude Fat (%):25.69 ± 0.12; Crude Fibre (%):9.96 ± 0.70,Crude Ash: 8.27 ± 0.07; Fish meal: Crude Protein(%): 72 ± 0.02,Crude Fat (%):15.6 ± 0.01,Crude Ash (%):14.5 ± 0.03. Trace mineral premix (1g/kg diet) were provided below: Manganese (Mn)- 115 mg; Zinc (Zn)- 88 mg; Copper (Cu)- 4 mg; Iron (Fe)- 44 mg; Iodine (I)-1.9 mg; Selenium (Se)-0.3 mg; Cobalt (Co)~0.05 mg. Vitamin mineral premix (IU/kg or mcg/kg or mg/kg diet) provided per kilogram of diet: Vitamin A: ~ 7,284 IU; Vitamin D₃: ~ 476 IU; Vitamin E: ~ 773 IU; Vitamin K (menadione): ~ 12 mg; Thiamine (B₁): ~ 44 mg; Riboflavin (B₂): ~ 56 mg; Niacin (B₃): ~ 420 mg; Pyridoxine (B₆): ~ 40 mg; Pantothenic acid: ~ 112 mg; Folic acid: ~ 9 mg; Biotin: ~ 0.42 mg; Vitamin B₁₂: ~ 23 µg; Vitamin C (ascorbic acid): ~ 412 mg; Manganese (Mn) −115 mg; Zinc (Zn) – 92 mg; Copper (Cu)- 5 mg; Iron (Fe) – 54 mg; Iodine (I) 1.8 mg; Selenium (Se)- 0.3 mg; Cobalt (Co) ~0.05 mg.

### Water quality parameters

The parameters of water quality analyzed are shown in Table 3. Significant differences existed in the water quality parameter during the different weeks, attributed to a disturbance due to the rains experienced during the trial period. Nevertheless, there wasn’t a significant difference in water pH (*P* = 0.5386). Additionally, the levels of nutrients, PO_4,_ N-NH_4_ and N-NO_3,_ in the water, and the corresponding phosphorus and nitrogen concentration per litre of water, varied weekly (*P* = 0.001) throughout the trial period, as shown in Table 3. This variation, however, did not adversely affect on the survival and growth of the experimental fish since the measurements were in accordance with the recommended range for African catfish.

**Table 3:** Water quality parameters in the experimental cages throughout the study period.

	Cage water physical attributes						Cage water nutrient attributes	
Week	pH	Conductivity	Temp ˚C	TDS*	Dissolved oxygen mg/l	Salinity	PO_4_ mg of P/l	N-NH_4_ mg of N/l
2	8.58 ± 0.10	104.00 ± 0.77^b^	27.40 ± 0.23^b^	51.3 ± 0.65^b^	10.20 ± 0.46^ba^	0.05 ± 0.001^b^	1.84 ± 0.37^dc^	0.08 ± 0.02^e^
4	8.38 ± 0.10	120.30 ± 0.77^a^	27.4 ± 0.23^b^	60.1 ± 0.65^a^	9.55 ± 0.46^ba^	0.06 ± 0.001^a^	3.04 ± 0.37^bac^	0.12 ± 0.02^ecd^
6	8.34 ± 0.10	121.00 ± 0.77^a^	28.3 ± 0.23^b^	60.5 ± 0.65^a^	9.81 ± 0.46^ba^	0.06 ± 0.001^a^	2.37 ± 0.37^bdc^	0.12 ± 0.022^ecd^
8	8.42 ± 0.10	122.00 ± 0.77^a^	28.1 ± 0.23^b^	61.0 ± 0.65^a^	9.07 ± 0.46^b^	0.06 ± 0.001^a^	2.64 ± 0.37^bdc^	0.18 ± 0.02^bcd^
10	8.58 ± 0.10	119.30 ± 0.77^a^	25.0 ± 0.23^d^	59.5 ± 0.65^a^	9.00 ± 0.46^b^	0.05 ± 0.001^b^	2.44 ± 0.37^bdc^	0.10 ± 0.02^ed^
12	8.59 ± 0.10	85.50 ± 0.77^d^	29.6 ± 0.23^a^	43.0 ± 0.65^c^	9.73 ± 0.46^ba^	0.04 ± 0.001^c^	2.97 ± 0.37^bdc^	0.16 ± 0.02^becd^
14	8.55 ± 0.10	99.00 ± 0.77^c^	25.1 ± 0.23^dc^	49.5 ± 0.65^b^	9.26 ± 0.46^b^	0.04 ± 0.001^c^	4.04 ± 0.37^ba^	0.28 ± 0.02^a^
16	8.36 ± 0.10	84.70 ± 0.77^d^	25.1 ± 0.23^dc^	42.0 ± 0.65^c^	9.53 ± 0.46^ba^	0.04 ± 0.001^c^	1.24 ± 0.37^d^	0.19 ± 0.02^bc^
18	8.36 ± 0.10	121.00 ± 0.77^a^	28.3 ± 0.23^b^	59.0 ± 0.65^a^	11.6 ± 0.46^a^	0.06 ± 0.001^a^	2.77 ± 0.37^bdc^	0.14 ± 0.02^becd^
20	8.39 ± 0.10	120.00 ± 0.77^a^	28.2 ± 0.23^b^	60.2 ± 0.65^a^	11.0 ± 0.46^ba^	0.06 ± 0.001^a^	3.04 ± 0.37^bac^	0.12 ± 0.02^ecd^
22	8.42 ± 0.10	100.30 ± 0.77^cb^	26.1 ± 0.23^c^	49.5 ± 0.65^b^	9.20 ± 0.46^b^	0.04 ± 0.001^c^	4.77 ± 0.37^a^	0.19 ± 0.02^bc^
24	8.43 ± 0.10	83.70 ± 0.77^d^	25.9 ± 0.23^dc^	42.0 ± 0.65^c^	9.29 ± 0.46^b^	0.04 ± 0.001^c^	2.24 ± 0.37^dc^	0.22 ± 0.02^ba^
F-value	0.91	425.70	45.80	147.40	2.97	35.40	6.26	12.30
P-value	0.539	0.001	0.001	0.001	0.0033	0.001	0.001	0.001

*TDS-Total dissolved solids expressed in mg/l. In every row, mean ± SE (n = 3) featuring same lower case superscripts letters are not significantly different (*P* < 0.05).

### Growth and survival of catfish fed on various diets

The growth performance indices, daily weight gain, SGR, FCR and survival rates are shown in Table 4. The initial mean lengths and weights of the fish in the different treatments were not significantly different (*P* > 0.05). There were, however, significant differences in the final mean weights and mean lengths (*P* < 0.05), depicting variations in the fish growth performance under different diets. Fish presented with D2 exhibited the highest daily weight gain (1.01 ± 0.02 g/day), lowest FCR (1.56 ± 0.10), highest SGR (2.15 ± 0.03) and a survival rate of 100%, recording the best growth performance. On the flip side, fish fed on diet C expressed the lowest daily weight gain (0.55 ± 0.02 g/day), highest FCR (3.74 ± 0.10), and lowest SGR (1.91 ± 0.03), recording the worst growth performance among the diets. Apart from the survival rates (*P* = 0.171), the daily weight gain (*P* = 0.001), FCR (*P* = 0.001) and SGR (*P* = 0.001) significantly varied among the different treatments.

**Table 4:** Growth performance indices of African catfish fed on experimental diets throughout the study.

Parameter	Experimental diets^1^						
	C	D1	D2	D3	D4	*F*-value	*P*-value
Initial weight(g)	3.44 ± 0.20	3.58 ± 0.20	4.18 ± 0.20	4.01 ± 0.20	3.50 ± 0.20	2.70	0.092
Initial length(cm)	8.36 ± 0.15	8.35 ± 0.15	8.75 ± 0.15	8.62 ± 0.15	8.25 ± 0.15	2.07	0.160
Final weight(g)	84.80 ± 2.79^b^	90.90 ± 2.79^b^	154.60 ± 2.79^a^	149.40 ± 2.79^a^	92.00 ± 2.79^b^	153.10	0.001
Final length(cm)	24.40 ± 0.93^bc^	25.10 ± 0.93^bac^	29.10 ± 0.93^a^	27.60 ± 0.93^ba^	22.80 ± 0.93^c^	7.51	0.005
Overall weight gain(g)	81.40 ± 2.78^b^	87.30 ± 2.78^b^	150.40 ± 2.78^a^	145.40 ± 2.78^a^	88.50 ± 2.78^b^	151.80	0.001
Overall length gain(cm)	16.10 ± 0.96^ba^	16.70 ± 0.96^ba^	20.40 ± 0.96^a^	19.00 ± 0.96^ba^	14.50 ± 0.96^b^	5.94	0.010
Daily weight gain(g)	0.55 ± 0.02^b^	0.59 ± 0.02^b^	1.01 ± 0.02^a^	0.98 ± 0.02^a^	0.59 ± 0.02^b^	151.80	0.001
Daily length gain(cm)	0.12 ± 0.01^ba^	0.11 ± 0.01^ba^	0.14 ± 0.01^a^	0.13 ± 0.01^ba^	0.10 ± 0.01^b^	5.94	0.010
FCR^2^	3.74 ± 0.10^a^	3.42 ± 0.10^a^	1.56 ± 0.10^b^	1.65 ± 0.10^b^	2.95 ± 0.10^a^	107.40	0.001
SGR%^3^	1.91 ± 0.03^b^	1.93 ± 0.03^b^	2.15 ± 0.03^a^	2.16 ± 0.03^a^	1.95 ± 0.03^b^	13.90	0.001
Survival %	98.30	96.70	100.00	100.00	100.00	2.00	0.171

^1^Experimental diets: 0% BSFLM and 100% FM (C – control diet), 25% BSFLM and 75% FM (D1), 50% BSFLM and 50% FM (D2), 75% BSFLM and 25% FM (D3), and 100% BSFLM and 0% FM (D4). Within rows, mean ± SD (n = 3) featuring same lower case superscripts letters are not significantly different (*P* < 0.05); 2FCR- feed conversion ratio; 3SGR-specific growth rate.

**Fig 1:**
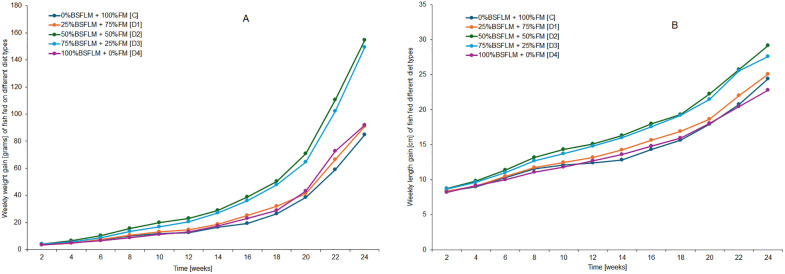
Weekly weight and length gain of catfish fed on the different diets.

Weight gain progression was observed throughout the trial period, depicting fish growth under the five different diets as presented in Fig 1. The figure shows that D2 was the best performing diet as compared to the others.

### Catfish fillet nutritional quality

The proximate composition of fish fillets per diet are shown in Table 5. Dry matter content was highest in D2 fillet (92.50 ± 0.73) and lowest in D1 fillet (90.20 ± 0.73), with no significant difference among the fillets from all diets (*P* = 0.147). Crude protein levels were highest in D2 fillet (75.00 ± 0.92) and lowest in D4 fillet (65.20 ± 0.92). Crude fat content was highest in D4 fillet (28.90 ± 1.52) and lowest in C fillet (15.60 ± 1.52), and total ash content was highest in D1 fillet (16.50 ± 0.56) and lowest in D2 (13.70 ± 0.56) and D3 (13.70 ± 0.56) fillets. Crude protein, crude fat and total ash content significantly (*P* < 0.05) varied among the fillets of fish fed on the five different diets.

**Table 5:** Proximate composition (g/kg of fish; wet weight basis) of catfish fillets from fish fed on experimental diets (mean ± SD; n = 3) on termination of the 168-day experimental period.

^1^Experimental diets	Dry matter	Crude protein	Crude fat	Total ash
C	90.40 ± 0.73	67.30 ± 0.92^b^	15.60 ± 1.52^b^	16.40 ± 0.56^a^
D1	90.20 ± 0.73	67.30 ± 0.92^b^	22.40 ± 1.52^ba^	16.50 ± 0.56^a^
D2	92.50 ± 0.73	75.00 ± 0.92^a^	25.00 ± 1.52^a^	13.70 ± 0.56^b^
D3	92.40 ± 0.73	72.20 ± 0.92^a^	26.60 ± 1.52^a^	13.70 ± 0.56^b^
D4	91.30 ± 0.73	65.20 ± 0.92^b^	28.90 ± 1.52^a^	15.50 ± 0.56^ba^
F-value	2.16	19.40	11.20	6.01
*P*-value	0.147	0.001	0.001	0.010

^1^Experimental diets: 0% BSFLM and 100% FM (C – control diet), 25% BSFLM and 75% FM (D1), 50% BSFLM and 50% FM (D2), 75% BSFLM and 25% FM (D3), and 100% BSFLM and 0% FM (D4); Within every row, mean ± SD (n = 3) featuring same lower case superscripts letters are not significantly different (*P* < 0.05).

The essential amino acid profiles of fish fillets per diet are shown Table 6. Indispensable amino acid levels significantly varied among the fish raised on the experimental diets (*P* < 0.05). All the indispensable amino acids levels were highest in fish fed on D2 (except arginine which was highest in D3-fed fish), and lowest in C diets-fed fish.

**Table 6:** Essential amino acid composition (*µ*g/g) of fish; wet weight basis) of African catfish fillets fed on experimental diets (mean ± SD; n = 3) on termination of the 168-day experimental period.

Amino acid	Treatments^1^						
	C	D1	D2	D3	D4	*F-value*	*P-value*
Arginine	11.40 ± 1.01^c^	21.70 ± 1.01^b^	33.10 ± 1.01^a^	34.10 ± 1.01^a^	24.80 ± 1.01^b^	85.20	0.001
Histidine	10.10 ± 0.55^c^	20.10 ± 0.55^b^	32.30 ± 0.55^a^	32.20 ± 0.55^a^	22.60 ± 0.55^b^	288.40	0.001
Isoleucine	7.38 ± 0.81^c^	15.20 ± 0.81^b^	20.20 ± 0.81^a^	17.70 ± 0.81^ba^	15.90 ± 0.81^b^	35.50	0.001
Leucine	11.70 ± 0.73^d^	17.30 ± 0.73^c^	22.20 ± 0.73^a^	21.90 ± 0.73^ba^	18.60 ± 0.73^bc^	34.40	0.001
Lysine	13.70 ± 0.79^c^	24.40 ± 0.79^b^	30.40 ± 0.79^a^	28.90 ± 0.79^a^	25.20 ± 0.79^b^	68.10	0.001
Methionine	5.83 ± 1.33^c^	16.60 ± 1.33^b^	33.30 ± 1.33^a^	31.20 ± 1.33^a^	30.90 ± 1.33^a^	80.70	0.001
Threonine	7.90 ± 0.88^d^	12.20 ± 0.88^c^	26.30 ± 0.88^a^	25.20 ± 0.88^a^	18.90 ± 0.88^b^	83.20	0.001
Phenylalanine	6.60 ± 0.67^c^	20.60 ± 0.67^b^	28.60 ± 0.67^a^	27.80 ± 0.67^a^	21.60 ± 0.67^b^	173.10	0.001
Valine	10.80 ± 0.85^c^	14.40 ± 0.85^bc^	19.50 ± 0.85^a^	18.70 ± 0.85^a^	16.30 ± 0.85^ba^	17.27	0.001

0% BSFLM and 100% FM (C – control diet), 25% BSFLM and 75% FM (D1), 50% BSFLM and 50% FM (D2), 75% BSFLM and 25% FM (D3), and 100% BSFLM and 0% FM (D4). Within rows, mean ± SD (n = 3) featuring same lower case superscripts letters are not significantly different (*P* < 0.05).

**Fig 2:**
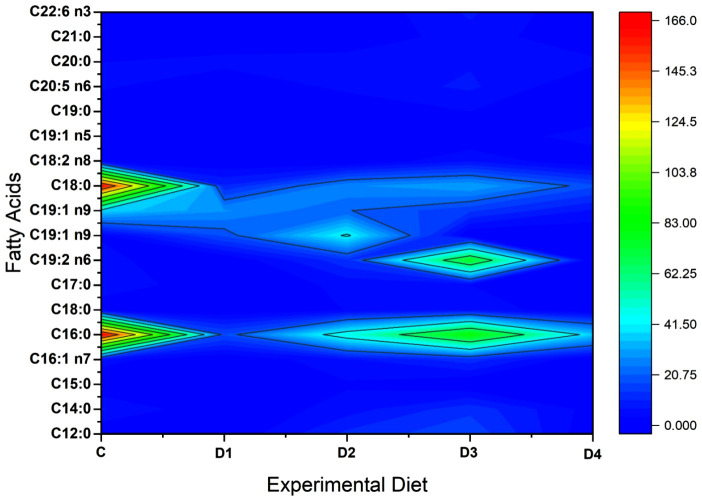
Fatty acids spectra of African catfish fillet when fed on diets supplemented with black soldier fly larvae meal.

Further, the fatty acids composition and spectra of the fillets from fish fed on the various experimental diets are represented in Table 7 and Fig 2. The polyunsaturated fatty acids (PUFAs), saturated fatty acids (SFAs) and monounsaturated fatty acids (MUFAs), were detected in most of the treatments. D3 displayed the highest number of detectable PUFAs with high abundance compared to the other samples. Lauric acid, the most abundant SFA of the medium chain fatty acids has been expressed highly in D3 and D2. In comparison to other treatments, D3 showed a 19-fold increase in linoleic acid levels. Among the treatments, the maximum range of SFA, MUFA and PUFA were seen in the order of D3 followed by D2, D1 and C. The ω-6/ω-3 index of the fish fed on C was 1.1/1, and 2.8/1 and 5/1 for D2 and D3, respectively.

**Table 7:** Fatty acid composition (µg/g of fish; wet weight basis) of catfish fillets fed on experimental diets (mean ± SD; n = 3) on termination of the 168-day experimental period.

		^*1*^ *Experimental diets*				
Rt	Library/ID	C	D1	D2	D3	D4
18.94	Methyl dodecanoate C12:0 (lauric acid)	–	–	11.4 ± 0.27^a^	16.7 ± 7.88^a^	0.2 ± 0.02^a^
21.25	Methyl tetradecanoate C14:0 (myristic acid)	7.3 ± 0.59^b^	0.02 ± 0.003^c^	5.2 ± 1.76^b^	12.8 ± 4.31^a^	1.9 ± 0.22^c^
22.30	Methyl pentadecanoate C15:0 (pentadecanoic acid)	0.2 ± 0.06^a^	0.002 ± .001^a^	3.0 ± 0.37^a^	–	–
23.13	Methyl 9*Z*-hexadecenoate C16:1 n7(palmitoleic acid)	5.0 ± 1.66^a^	–	6.4 ± 2.39^a^	12.4 ± 4.32^a^	–
23.33	Methyl hexadecanoate C16:0 (palmitic acid)	165.7 ± 3.80^b^	17.7 ± 4.54^a^	47.3 ± 12.55^a^	82.5 ± 23.08^a^	36.5 ± 2.16^a^
24.02	Methyl 14-methylhexadecanoate C18:0 (anteiso-Heptadecanoic acid)	–		3.6 ± 1.05^a^	6.2 ± 0.38^a^	–
24.29	Methyl heptadecanoate C17:0 (margaric acid)	6.6 ± 0.02^a^	0.01 ± 0.002^a^	4.5 ± 0.27^a^	3.5 ± 0.76^a^	–
24.94	Methyl (9*Z*,12*Z*)-octadecadienoate C19:2 n6 (linoleic acid)	4.6 ± 1.52^a^	0.02 ± 0.002^a^	13.0 ± 3.51^a^	76.1 ± 33.08^a^	–
24.98	Methyl 9*E*-Octadecenoate C19:1 n9 (elaidic acid)	3.1 ± 1.03^a^	18.0 ± 0.68^a^	42.7 ± 4.35^a^	0.02 ± 0.001^a^	–
24.99	Methyl 9*Z*-Octadecenoate C19:1 n9 (oleic acid)	37.6 ± 12.53^a^	28.2 ± 3.58^a^	21.0 ± 1.51^a^	14.6 ± 0.60^a^	6.4 ± 0.26^a^
25.23	Methyl octadecenoate C18:0 (stearic acid)	163.1 ± 3.07^b^	10.6 ± 0.57^a^	27.2 ± 3.04^a^	31.2 ± 5.19^a^	18.2 ± 1.50^a^
25.34	Methyl 6,10-octadecadienoate C18:2 n8 (6,10-octadecadienoic acid)	–	–	–	6.5 ± 0.83	–
25.01	Methyl 13*E*-Octadecenoate C19:1 n5 (trans-13-Octadecenoic acid)	–	–	–		5.8 ± 0.17
26.11	Methyl nonadecanoate C19:0 (nonadecanoic acid)	–	–	–	3.5 ± 0.42^a^	–
26.44	Methyl (5*Z*,8*Z*,11*Z*,14*Z*)-Eicosapentaenoate C20:5 n3 (eicosapentaenoic acid; EPA)	4.2 ± 1.41^a^	–	4.6 ± 1.16^a^	7.6 ± 1.22^a^	–
26.98	Methyl eicosanoate C20:0 (arachidic acid)	3.4 ± 1.00^a^	4.9 ± 0.21^a^	5.8 ± 0.28^a^	6.5 ± 0.17^a^	4.4 ± 0.35^a^
26.98	Methyl 18-methylnonadecanoate C21:0 (isoarachidic acid)	–	–	–	5.7 ± 0.19	–
28.05	Methyl (4*Z*,7*Z*,10*Z*,13*Z*,16*Z*,19*Z*)-Docosahexaenoate C22:6 n3 (docosahexaenoic acid; DHA)	–	–	–	7.5 ± 2.19	–
	∑SFA	351.2 ± 6.03^b^	33.4 ± 3.48^a^	109.7 ± 5.71^b^	171.3 ± 6.02^b^	61.8 ± 4.16^a^
	∑MUFA	46.8 ± 3.62^a^	48.1 ± 4.07^a^	72.2 ± 4.40^a^	27.1 ± 2.38^a^	12.5 ± 3.67^a^
	∑PUFA	8.9 ± 1.08^a^	0.02 ± 0.003^a^	18.1 ± 3.36^a^	99.8 ± 5.83^a^	–
	∑PUFA (n-3)	4.2 ± 1.41^a^	–	4.6 ± 1.16^a^	15.2 ± 2.52^a^	–
	∑PUFA (n-6)	4.6 ± 1.52^a^	0.02 ± 0.002^a^	13.0 ± 3.51^a^	76.1 ± 33.08^a^	–
	n-6/n-3	1.1	–	2.8	5	–

^1^Experimental diets: 0% BSFLM and 100% FM (C – control diet), 25% BSFLM and 75% FM (D1), 50% BSFLM and 50% FM (D2), 75% BSFLM and 25% FM (D3), and 100% BSFLM and 0% FM (D4)); ∑SFA = sum of all saturated fatty acids, ∑MUFA = sum of all monounsaturated fatty acids, ∑PUFA = sum of all polyunsaturated fatty acids; Mean ± SD (n = 3) bearing different superscripts within each row are significantly different (p < 0.05)

### Cost-benefit analysis of catfish production with BSFLM diets

The economic implications of substituting FM with BSFLM are shown in Table 8. The cost of production was highest in C and reduced with increase in the replacement levels of BSFLM, with a significant variation across the diets. Gross profit (USD 9.02), gross profit margin (4.63), CBR (1.05) and RoI (105.2%) were highest in D2 and lowest in control C, with a significant variation across the diets.

**Table 8:** Cost-benefit analysis of African catfish production with black soldier fly larvae meal diets.

^1^Experimental diet	Cumulative intake (kg)	Production costs (USD)	Gross profit (USD)	Gross profit margin	^2^CBR	^3^RoI
C	5.43 ± 0.12	4.45^a^	4.88^e^	0.43^e^	0.10^e^	9.69 ± 0.40^e^
D1	5.43 ± 0.12	4.42^b^	5.24^d^	0.81^d^	0.18^d^	18.40 ± 0.40^d^
D2	5.43 ± 0.12	4.40^c^	9.02^a^	4.63^a^	1.05a	105.20 ± 0.40^a^
D3	5.43 ± 0.12	4.37^d^	8.72^b^	4.36^b^	1.00^b^	99.70 ± 0.40^b^
D4	5.43 ± 0.12	4.32^e^	5.31^d^	0.99^c^	0.23^c^	22.90 ± 0.40^d^
*F value*	0.22	*	*	*	*	14397.2
*P* – value	0.921	<0.001	<0.001	<0.001	<0.001	<0.001

^1^Experimental diets: 0% BSFLM and 100% FM (C – control diet), 25% BSFLM and 75% FM (D1), 50% BSFLM and 50% FM (D2), 75% BSFLM and 25% FM (D3), and 100% BSFLM and 0% FM (D4); ^2^CBR = Cost Benefit Ratio; ^3^RoI = Return on Investment. Within rows, mean ± SD (n = 3) featuring same lower case superscripts letters are not significantly different (*P* < 0.05).

## Discussion

The need for addressing deficiencies of quality and sustainable animal proteins for livestock and human consumption in Sub-Saharan Africa calls for local, sustainable production and utilization of ingredients [2]. It is well understood that Sub-Saharan Africa populace and the global south at large is generally protein deficient [62–65], and new study has found that smallholder farms play a vital role in reducing this challenge. Several investigations have alluded to the fact that insect-derived meals are notably high in protein and could serve as a pivotal solution to address malnutrition and deficiencies of protein. This is very pertinent in today’s context due to the substantial competition between humans and animals for FM, resulting in increased market prices [66]. In Kenya, smallholder farms are increasingly adopting the rearing of BSFL as a sustainable practice to feed and support various livestock species [67]. To the full extent of our understanding, this study is the first to have comprehensively investigated the impact of fully fatted BSFLM-based aquafeed on the growth performance, fillet quality and cost-effectiveness of *C. gariepinus* production in outdoor cage-in-pond culture system for a prolonged culture duration.

The quality of the BSFLM currently employed in this study aligns with that utilized in previous studies [22,48,59,60], as it was sourced from the same facility. In addition, the proximate contents of the diets derived from the BSFLM and FM combinations were within the recommended nutritional requirements of the *C. gariepinus* [68]. *C. gariepinus* is one of the few fish species able to breathe air, making it a versatile fish even where water contamination is a concern [69–71]. The cage water quality parameters met water quality requirements for African catfish rearing [72–74]. The authors attribute the variation in water quality indices as an effect of the seasonal rains experienced in the region during the study period. The cages installed in the pond had a feeder canal from a larger river flowing from Mt. Kenya Forest. Although the water parameters were changing every other week, the observable health of the experimental fish was consistently unaffected. African catfish can survive and reproduce in murky waters with highly dissolved solids, low oxygen, and debris [75–77]. Ammonia levels of over 0.2 mg/l have been reported to be toxic to the channel catfish [78], whereas Toko et al. [79] and Okomoda et al. [80] reported that dissolved oxygen levels of between 0.9 and 1.2 ppm were still ideal to allow the African catfish to grow normally and reproduce. Despite the rains experienced during this study, there was little to no siltation or water contamination warranting concern that could adversely affect the water quality.

The study demonstrated a significant effect of partially substituting FM with BSFLM on the feed utilization and growth performance of *C. gariepinus*. Notably, substituting FM with BSFLM up to the share of 50% yielded excellent growth performance and feed utilization of *C. gariepinus*, followed by an inclusion of 75% BSFLM. These findings coincide with previous studies, indicating that the best growth performance occurs within the range of 50–75% BSFLM inclusion in the culture of a number of fish species, such as *C. gariepinus* [44,47], *Oreochromis niloticus* [48], and *S. salar* [12] This is attributed to synergetic dietary energy interactions between the two protein sources (FM and BSFLM) at these levels [81]. The improved FCR and SGR in BSFLM incorporated diets at 50 and 75% inclusion levels suggests that incorporating BSFLM in the feed has the potential to augment palatability, thereby contributing to an elevated growth rate and final weight of the fish. Also, due to its carnivory, the availability of pyloric caeca in *C. gariepinus* with less abundance of secretory glands and the presence of a thick tunica muscularis facilitates efficient digestion even up to 75% BSFLM incorporated diets [82]. The low growth performance in the 100% FM diet can be attributed to oxidative degradation since the amino acid profile of FM may not always be optimal, and it is prone to lipid oxidation, leading to rancidity and potential growth suppression [29]. In contrast, BSFLM contains bioactive compounds such as antimicrobial peptides and lauric acid, which enhance gut health and nutrient absorption, contributing to improved feed conversion efficiency [29,44]. Furthermore, BSFLM has been shown to enhance fish immunity, reducing energy expenditure on immune responses and promoting better overall growth [40].

However, the observed decline in growth performance in the 100% BSFLM incorporated diet could be linked to reduced feed intake and a rejection of the diet by the fish. This consequently results in a diminished intake of essential protein and energy needed for growth of the African catfish. This observation is in accordance with prior investigation that has reported decreased palatability and reduced feed intake in instances where FM is partially or entirely substituted in fish diets [83].

The current findings align with earlier research that used whole, partially defatted, or fully defatted BSFLM [23,32,37,84,85]. Previous studies investigating FM replacement with BSFL in fish diets [22,29,38,40,44,48,86,87] consistently reported no detrimental effects on the survival rates of the fish species. This observation concorded with the current study results, where no significant variations in survival rates were witnessed across the various experimental groups.

The current study demonstrated that the different dietary treatments affect African catfish fillet proximate composition. The dietary replacement of BSFLM influenced two essential nutrients, crude protein and crude fat. Fillet protein displayed a positive correlation with increasing levels of BSFLM replacement, reaching its peak at 75% replacement level. Nevertheless, surpassing this threshold resulted in a discernible decline in protein levels. This is because of protein-sparing action by the high-fat content supplied by growing levels of BSFLM [88,89]. Conversely, reduced protein may be associated with reduced dietary intake due to higher dietary energy [90]. In contrast to the present findings, a prior investigation by Katya et al. [91] found that the substitution of BSFLM did not influence carcass compositions of juvenile Barramundi (*Lates calcarifer*). Other investigations have also discovered insignificant differences in proximate contents, particularly regarding the share of crude protein of trout [25,28]. However, Zarantoniello et al. [92] reported an enhancement of the total fatty acids in zebrafish fed with 50% BSFLM diet. The current study revealed that including full fat BSFLM increased total crude fat, which may directly translate to an enhancement in fatty acids, particularly omega 6 fatty acids. This increase could potentiate an increase in dietary omega-3 sourced from farmed African catfish reared on full fat BSFLM.

The amino acid composition of *C. gariepinus* decreased with escalating BSFLM levels. Incorporation of BSFLM up to 75% in feed showcased the expression of EAAs surpassing the nutritional requirements of African catfish [93]. Furthermore, the observed decline in growth within the 100% BSFLM diet could be attributed to a relative decrease in methionine in the diet, which is a limiting EAA in BSFLM [94,95]. As indicated by Xiao et al. [40], insufficient levels of lysine or methionine have been linked to reduced fish growth and feed efficiency. Consequently, considering methionine supplemented BSFLM diet may be a prudent approach to overcome the growth retardation.

Fish fatty acid profiles appear to parallel the profiles of the diet. The major fatty acid constituents of 75% BSFLM diet reared *C. gariepinus* fillet had a range of PUFAs (fats that contains more than one double bond in their chemical structure), MUFAs (fats that contain one double bond in their chemical structure) and SFAs (fats that have no double bonds in their chemical structure, meaning they are fully “saturated” with hydrogen atoms) due to the increased crude fat. The abundant medium chain fatty acid, lauric acid, displayed elevated levels in 50% and 75% BSFLM diets. This observation may be ascribed to the prevalence of lauric acid as the primary lipid in black soldier fly larvae, forming a melanin–chitosan complex known for its extensive antibacterial activity [96]. The enhanced proportion of lauric acid in the larvae positively influences feed intake, nutrient absorption, gut development, and subsequently contributes to the enhanced growth of the fish [97]. These findings explain the higher growth rates observed in 50% and 75% BSFLM diets.

Linoleic acid in 75% BSFLM diet showed 19-fold increase from the control diet, and this corroborates with the increased DHA and EPA levels as compared to other diets. In freshwater fish, linoleic acid is crucial for growth, survival, and the functioning of cells [98]. Unlike marine fish, freshwater fish possess the unique ability to convert linoleic acid into vital fatty acids such as arachidonic acid, DHA, and EPA [99]. Linoleic acid is a crucial omega-6 unsaturated fatty acid requisite for proper development and growth [100]. It also serves as a key precursor to arachidonic acid, which contributes to neuron development and other physiological functions [101]. These results suggest that incorporating up to 75% BSFLM in diets of African catfish can markedly enhance their nutritional value.

The present study reinforces reports from other studies asserting that the fatty acid spectrum of fish fed on diet integrated with full-fat BSFLM performs better than the fish raised on FM-incorporated diets. Also, the current findings contradict previous reports indicating that the deficiencies of indispensable fatty acids, particularly DHA and EPA in BSFLM, as the research outcomes may differ based on the insect species, substrate composition and insect processing methodologies as well as the size and species of the fish [8]. The incorporation of elevated levels of BSFLM in *C. gariepinus* resulted in a reduction of MUFA and PUFA contents, as evidenced by a prior study conducted by Zarantoniello et al. [92]. The witnessed variations in the fatty acid spectra and crude fat contents of the fish fed with BSFLM may be intricately linked to the substrate properties utilized for BSFL rearing and the dietary preferences inherent to the dietary patterns of the fish [102].

Polyunsaturated fatty acids (PUFAs) from the ω-3 and ω-6 groups are crucial for human health [103]. Key PUFAs include linoleate, dihomo-γ-linolenate and arachidonate of the ω-6 category, and ALA, DHA, and EPA from the ω-3 category [104]. These PUFAs are involved in regulating various bodily functions, such as pain, sleep, immunity, cell growth, inflammation, platelet activity, and neural plasticity. Generally, ω-3 PUFAs reduce platelet aggregation and inflammation while promoting vasodilation, whereas ω-6 PUFAs tend to enhance platelet aggregation, inflammation, and vasoconstriction [105]. Although both the ω-6 and ω-3 fatty acids contribute to positive health outcomes, their reversing metabolic effects can lead to disease-related processes in the body [106]. Therefore, maintaining an appropriate ω-6/ω-3 index in the diet is essential. The recommended intake ratio is 4–5/1 or lower [106,107]. In our study, the n-6/n-3 index in fish fed BSFL diets ranged from 2.8/1–5/1, which fell within the recommended range. This suggests that incorporating 50–75% BSFL into fish diets improves their PUFA content and overall nutritional quality.

In the current study, the highest production cost, attributed to feed expenses, was realized in the control diet, which was reduced progressively as BSFLM incorporation was increased. It has been documented that FM replacement by BSFLM reduces feed costs by up to 40% hence enhancing profitability in the rearing of *O. niloticus* [48] and *Dicentrarchus labrax* [108]. Onsongo et al. [16], Sumbule et al. [59] and Waithaka et al. [60], while rearing broilers, layers and indigenous chicken, respectively, using BSFLM, found that the feed cost was reduced compared to the conventional diet. Economic efficiencies, such as increased profit margins and percentage return on investments realized in the current study, especially with partial replacements of FM with 50–75% BSFLM, mirror previous studies using *O. niloticus* [109,110]. Chia et al. [111] reported that the favourable economics of adopting BSFLM as a substitute source of protein enhances sustainability and could therefore reduce the nutrient deficiencies in smallholder livestock farms in sub-Saharan Africa. At a commercial scale, using BSFLM can reduce reliance on costly imported conventional protein sources, thereby strengthening local feed industry resilience and improving farmers’ income stability.

The BSF farming industry is thriving at a swift pace, and the use of BSFLM as an aquafeed ingredient would positively and substantially impact aquaculture practices, making aquaculture green, productive, and sustainable. Compared to crop-based proteins, BSFLM production reduces the need for agricultural land expansion, helping to curb deforestation and habitat loss, while facilitating nutrient recovery from agro-industrial by-products to close nutrient loops and reduce eutrophication risks [112]. However, large-scale production of BSFLM also presents potential environmental trade-offs that must be considered, including the energy demand for maintaining optimal rearing temperatures, emissions from substrate processing, and water use for larval cleaning and drying. Life cycle assessment (LCA) studies have shown that BSFLM production emits between 2.2 and 5.1 kg CO₂-equivalent per kg of dry product, which is significantly lower than fishmeal, estimated at 4.9 to 6.4 kg CO₂-eq/kg [112–114]. Additionally, BSFLM requires less land (0.2–0.6 m²/kg) and water (approximately 1,000–1,800 Litres/kg) compared to soybean meal or fishmeal [115]. Despite these, variations in feedstock quality, processing methods, and energy sources can influence the overall sustainability. Therefore, conducting region-specific LCAs and adopting low-impact processing technologies are critical to fully realizing the environmental benefits of BSFLM, position it as a sustainable alternative protein source for aquaculture.

## Conclusions

The current study proved that 50 and 70% substitution of FM with BSFLM significantly enhanced the growth performance of African catfish as compared to other inclusion levels. Elevating the replacement of BSFLM beyond these levels did not yield further improvements in growth performance. Additionally, the fillet nutritional quality of African catfish reared on BSFLM showed superior quality, including indispensable amino acids and fatty acids (as evidenced by 19-fold increase in linoleic acid) content. These feeding trials have shown that replacement levels of 50–75% BSFLM are recommended for the Sagana strain of African catfish, but higher replacement levels of up to 100% have detrimental effects, compromising the growth performance and nutritional quality of the fish. Furthermore, the investigation identifies a noteworthy impact on economic performance, attributed to the comparative cost-effectiveness of BSFLM protein in relation to FM.

From this study, it can be deduced that 50–75% replacement of FM with BSFLM in *C. gariepinus* diets results in enhanced growth performance, fillet quality and economic viability. Therefore, integration of BSFLM in aquafeed can significantly reduce competition with traditional protein sources, due to their comparative advantages in nutritional value. Considering this, further research is inevitable to optimize the nutritional potential of BSFLM for catfish diets. This should consider various factors, such as its mineral content, chemical composition, amino acid profiles (especially lysine and methionine), nutrient and energy bioavailability, fatty acids, and water-soluble vitamins. Additionally, it is essential to refine the processes used to produce diets that meet the specific nutritional needs, digestibility, feeding preferences, and palatability of different aquaculture species. Since BSF efficiently converts biowaste into high value protein biomass, proper sanitation protocols must be established to ensure that BSFLM is free from pathogens and harmful contaminants for safe use.

While many East African countries have established production standards and guidelines for using BSFLM in aquafeeds, it is essential to develop a comprehensive legal framework and legislation, alongside enhancing risk assessment procedures at the continental level. Further investigation into the effects of feeding aquaculture species with BSFLM, particularly in terms of safety, quality, and societal acceptance of the fish, is urgently needed. The BSF farming industry is growing swiftly, and the use of BSFLM as aquafeed ingredient is promising as a sustainable alternative to conventional fish feed ingredients.

## Supporting information

S1 File Protocols for amino acid and fatty acid analyses. (PDF)
