# Supplementary material for: Emerging insect-based aquafeed for sustainable African catfish production
Source: PLoS One. 2025 Nov 26;20(11):e0335422. doi: 10.1371/journal.pone.0335422 (PMC12654937; doi:10.1371/journal.pone.0335422)
Supplement: S1 File — (PDF) [file pone.0335422.s001.pdf]

Protocol for Determination of Amino Acid and Fatty Acid Composition in Fish Fillets

A. Amino Acid Analysis by LC-MS

Objective
To hydrolyze proteins in fish fillet into free amino acids and quantify them using Liquid Chromatography-Mass Spectrometry (LC-MS).

Materials and Reagents

- Fish fillet powder (25 mg per sample)
- 6N Hydrochloric acid (HCl)
- Nitrogen gas
- Pyrex digestion tubes with Teflon-lined caps
- Oven (capable of 110 °C)
- 0.01% formic acid/acetonitrile (95:5 v/v)
- Vortex mixer
- Centrifuge
- LC-MS system
- LC-MS grade water, methanol, and acetonitrile
- Syringe filters (0.22 µm)

Procedure

1. Sample preparation
   - Weigh 25 mg of finely ground fish fillet into a clean Pyrex digestion tube.

2. Acid hydrolysis
   - Add 5 mL of 6N HCl to each tube.
   - Flush with nitrogen gas and tightly seal the tubes with Teflon-lined caps.

3. Hydrolysis
   - Place tubes in an oven at 110°C for 22 hours.

4. Drying the hydrolysate

- Cool, then evaporate to dryness using a vacuum concentrator or rotary evaporator.

5. Reconstitution
   - Reconstitute the dried hydrolysate with 1 mL of 0.01% formic acid/acetonitrile (95:5).
   - Vortex vigorously.

6. Centrifugation
   - Centrifuge at 10,000 rpm for 10 minutes and filter supernatant using a 0.22 µm filter.

7. LC-MS analysis
   - Inject 0.3 µL into the LC-MS.
   - Use a reverse-phase C18 column.
   - Mobile Phases:
     a) Water + 0.1% formic acid
     b) Acetonitrile + 0.1% formic acid
   - Run a gradient suitable for separating amino acids.
   - Identify and quantify using retention time and m/z ratio.

B. Fatty Acid Analysis by GC-MS

Objective
To convert fatty acids into volatile methyl esters (FAMEs) and quantify them using Gas Chromatography-Mass Spectrometry (GC-MS).

Materials and Reagents

- Fish fillet powder (100 mg per sample)
- Methanolic sodium methoxide solution (0.5 M)
- Hexane (HPLC grade)
- Anhydrous sodium sulfate
- Vortex mixer
- Centrifuge
- GC-MS system (e.g., Agilent 7890A GC with 5975C MS)
- GC capillary column (e.g., DB-23 or DB-Wax, 30 m × 0.25 mm × 0.25 µm)
- FAME standards

Procedure

1. Sample weighing
   - Weigh 100 mg of homogenized fish fillet into a glass vial.

2. Transmethylation
   - Add 2 mL of methanolic sodium methoxide (0.5 M).
   - Incubate at 70°C for 1 h with intermittent shaking.

3. Extraction of FAMEs
   - Cool and add 1 mL hexane.
   - Vortex for 1–2 minutes.

4. Phase separation
   - Centrifuge at 4,000 rpm for 5 minutes.
   - Collect the upper hexane layer.

5. Drying the extract
   - Pass through anhydrous sodium sulfate.

6. GC-MS analysis
   - Inject 1 µL into GC-MS.
   - Temperature Program:
     - Start: 50°C (1 min hold)
     - Ramp: 10°C/min to 250°C
     - Final hold: 5 min at 250°C
   - Carrier gas: Helium at 1 mL/min
   - Ionization mode: EI
   - Identify peaks using retention times and mass spectra vs. standards.

References

Musundire R, Osuga IM, Cheseto X, Irungu J, Torto B. Aflatoxin contamination detected in nutrient and anti-oxidant rich edible stink bug stored in recycled grain containers. PLoS One. 2016;11:1–16.

Ochieng BO, Anyango JO, Khamis FM, Ekesi S, Egonyu JP, Subramanian S, et al. Nutritional characteristics, microbial loads and consumer acceptability of cookies enriched with insect (*Ruspolia differens*) meal. LWT. 2023;184: 115012. doi:10.1016/J.LWT.2023.115012

Hamilton ML, Kuate SP, Brazier-Hicks M, Caulfield JC, Rose R, Edwards R, et al. Elucidation of the biosynthesis of the di-C-glycosylflavone isoschaftoside, an allelopathic component from Desmodium spp. that inhibits Striga spp. development. Phytochemistry. 2012 Dec 1;84:169–76.
